# Nuclear localization of heparanase 2 (Hpa2) attenuates breast carcinoma growth and metastasis

**DOI:** 10.1038/s41419-024-06596-8

**Published:** 2024-03-22

**Authors:** Maram Hilwi, Katherina Shulman, Inna Naroditsky, Sari Feld, Miriam Gross-Cohen, Ilanit Boyango, Soaad Soboh, Olga Vornicova, Malik Farhoud, Preeti Singh, Gil Bar-Sela, Hadassah Goldberg, Martin Götte, Andrew D. Sharrocks, Yaoyong Li, Ralph D. Sanderson, Neta Ilan, Israel Vlodavsky

**Affiliations:** 1 Technion Integrated Cancer Center, Rappaport Faculty of Medicine, Technion, Haifa, Israel; 2 Department of Oncology, Carmel Medical Center, Haifa, Israel; 3 Departments of Pathology, Rambam Health Care Campus, Haifa, Israel; 4 Department of Oncology, Ha’amek Medical Center, Afula, Israel; 5 Department of Gynecology and Obstetrics, Münster University Hospital, Muenster, Germany; 6 Faculty of Biology, Medicine and Health, University of Manchester, Manchester, UK; 7 Department of Pathology, University of Alabama at Birmingham, Birmingham, AL USA

**Keywords:** Breast cancer, Mechanisms of disease

## Abstract

Unlike the intense research effort devoted to exploring the significance of heparanase in cancer, very little attention was given to Hpa2, a close homolog of heparanase. Here, we explored the role of Hpa2 in breast cancer. Unexpectedly, we found that patients endowed with high levels of Hpa2 exhibited a higher incidence of tumor metastasis and survived less than patients with low levels of Hpa2. Immunohistochemical examination revealed that in normal breast tissue, Hpa2 localizes primarily in the cell nucleus. In striking contrast, in breast carcinoma, Hpa2 expression is not only decreased but also loses its nuclear localization and appears diffuse in the cell cytoplasm. Importantly, breast cancer patients in which nuclear localization of Hpa2 is retained exhibited reduced lymph-node metastasis, suggesting that nuclear localization of Hpa2 plays a protective role in breast cancer progression. To examine this possibility, we engineered a gene construct that directs Hpa2 to the cell nucleus (Hpa2-Nuc). Notably, overexpression of Hpa2 in breast carcinoma cells resulted in bigger tumors, whereas targeting Hpa2 to the cell nucleus attenuated tumor growth and tumor metastasis. RNAseq analysis was performed to reveal differentially expressed genes (DEG) in Hpa2-Nuc tumors vs. control. The analysis revealed, among others, decreased expression of genes associated with the hallmark of Kras, beta-catenin, and TNF-alpha (via NFkB) signaling. Our results imply that nuclear localization of Hpa2 prominently regulates gene transcription, resulting in attenuation of breast tumorigenesis. Thus, nuclear Hpa2 may be used as a predictive parameter in personalized medicine for breast cancer patients.

## Introduction

HPSE2, the gene encoding Heparanase 2 (Hpa2), was cloned shortly after the cloning of heparanase, based on sequence homology [1]. Hpa2, nonetheless, gained little attention, possibly because it fails to cleave heparan sulfate (HS) side chains of heparan sulfate proteoglycans (HSPG), the hallmark of heparanase enzymatic activity [2]. Hpa2, however, is not an inert protein but rather plays important roles in several human pathologies. This is best demonstrated by the finding that HPSE2 is mutated in urofacial syndrome (UFS), a rare autosomal recessive inherited disease [3, 4]. In UFS, biallelic mutations of HPSE2 mostly result in frameshifts that lead to an early stop codon and a truncated protein, resulting in Hpa2-null phenotype [3, 4]. The lack of Hpa2 appears to be responsible for peripheral neuropathy of the bladder, leading to incomplete bladder emptying and renal pelvis reflux of urine from the bladder to the upper renal tract, with a risk of kidney damage and renal failure [5, 6]. Neuropathy likely affects also facial nerves because people with UFS have a characteristic expression, like in pain, upon smiling [7], suggesting that Hpa2 plays an important role in neurons. Decreased levels of Hpa2 were noted in several other pathological conditions. For example, a prominent decrease in the levels of Hpa2 was quantified in the plasma of patients exhibiting severe symptoms of COVID-19 [8] and in conditions of sepsis [9]. Notably, administration of purified Hpa2 relieved the symptoms of sepsis in a mouse model [9]. Moreover, therapeutic plasma exchange (TPE) administrated to patients with early septic shock restored Hpa2 levels and protected the vascular glycocalyx [8], thus offering, possibly, therapeutic intervention for this life-threatening condition [10, 11]. The mechanism underlying the protective role of Hpa2 for vascular endothelial cells is not entirely clear but is thought to involve heparanase activity. This notion is based on the capacity of Hpa2 to inhibit heparanase enzymatic activity due to its high affinity to HS and competition for the substrate of heparanase, and/or physical association between heparanase and Hpa2 [2]. Thus, decreased levels of Hpa2 tilt the balance between heparanase and Hpa2 in favor of heparanase, resulting in increased heparanase activity. Elevation of heparanase activity, in turn, results in the destruction of the glycocalyx and disruption of the vasculature [8–12].

A similar scenario may also occur in cancer. Unlike heparanase, Hpa2 is readily detected in normal epithelium of the bladder, breast, cervical, gastric, and ovarian tissues but its expression is markedly decreased in the resulting carcinomas [13–18]. Remarkably, patients endowed with high levels of Hpa2 survived longer than patients showing low levels of Hpa2 [2, 16, 19–21]. In addition, over-expression of Hpa2 in cancer cell lines resulted in smaller tumor xenografts, whereas silencing of Hpa2 resulted in bigger tumors [2, 14, 16, 19, 22–24]. These results suggest that Hpa2 is critically important for epithelial cell integrity and functions as a tumor suppressor.

Here, we examined the role of Hpa2 in breast cancer. We describe, for the first time, that Hpa2 is localized predominantly in the nucleus of normal breast epithelium. In breast cancer, Hpa2 expression is not only decreased but also loses its nuclear localization and appears diffuse in the cell cytoplasm. Being a secreted protein, this ‘cytoplasmic’ staining mostly reflects the biosynthetic route of Hpa2, localizing to the ER and Golgi apparatuses. Surprisingly, breast cancer patients showing high levels of Hpa2 survived less than Hpa2-low patients and exhibited higher incidents of metastasis. In contrast, tumor metastasis was reduced in breast tumors in which nuclear localization of Hpa2 was retained. These results strongly imply that in breast cancer, cellular localization of Hpa2 plays an important role in the progression of the disease.

## Results

### High levels of Hpa2 are associated with bad prognosis of breast cancer patients

**Fig. 1:**
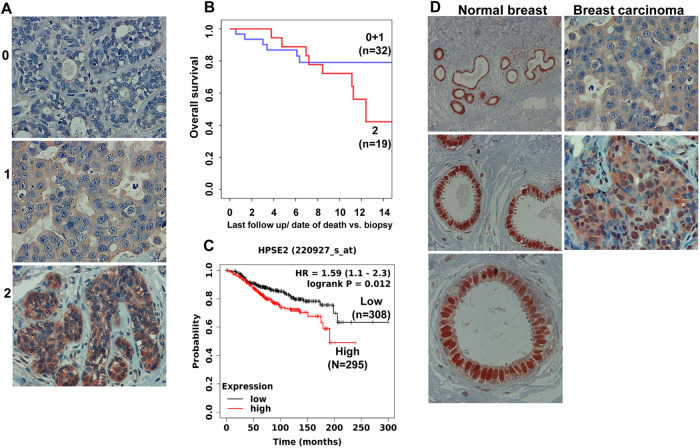
Hpa2 expression and localization in young breast carcinoma patients. **A** Immunostaining. Breast carcinoma specimens (*n* = 51) were subjected to immunostaining applying anti-Hpa2 antibody. The staining was examined by a senior pathologist and was scored as negative (0; *n* = 18), weak (1; *n* = 14), or strong (2; *n* = 19) staining intensity. Shown are representative images. Original magnuficatios: ×100. **B** and **C** Patient survival. **B** The staining intensity of Hpa2 (0 + 1; *n* = 32 vs. 2; *n* = 19) was correlated with the survival of the patients (*n* = 51) and presented as a Kaplan–Meier (KM) survival plot. **C** Levels of Hpa2 (low; *n* = 308 vs. high; *n* = 295) were retrieved from a large cohort (*n* = 603) of ER-positive, HER2-negative breast cancer patients, and survival analysis was calculated by a public server (KM plotter service; https://kmplot.com/analysis). Note shorter survival of breast carcinoma patients exhibiting high levels of Hpa2; *p* = 0.01. **D** Hpa2 is localized to the cell nucleus in normal breast epithelium. Immunostaining revealed that in normal breast tissue adjacent to the tumor lesion, Hpa2 appears to localize mainly to the cell nucleus (**D**, Normal breast). In breast carcinoma, Hpa2 staining intensity is prominently decreased in many cases and appears mainly diffused in the cell cytoplasm (**D**, upper right) or exhibiting cytoplasmic and nuclear staining (**D**, lower right). Shown are representative images at original magnifications of ×25 (upper left), ×100 (middle left and right panels), and ×250 (lower left).

Hpa2 expression was noted to be reduced in breast carcinoma vs normal breast tissue [13], but the role of Hpa2 in breast cancer has not been reported yet. To examine this aspect, we subjected a cohort of 61 breast carcinoma biopsies of young women (age 19–45, median 37.1; Supplementary Table 1) to immunostaining applying anti-Hpa2 antibody. Hpa2 staining was successfully obtained in 51 tumor biopsies. We found that 35% (18/51) of the biopsies were stained negative for Hpa2 (0), whereas 65% (33/51) were stained positive, exhibiting weak (+1; 14/48) or strong (+2; 19/48) staining (Fig. 1A). We next correlated the staining intensity with clinical parameters and found that high levels of Hpa2 correlate inversely with tumor grade (Table 1), in agreement with a similar correlation reported in bladder [14], pancreatic [19], and gastric [16, 21] carcinomas. Surprisingly, strong staining of Hpa2 was associated with increased incidence of lymph node metastasis (N-stage; Table 1) and shorter overall survival of breast cancer patients (Fig. 1B; *p* = 0.1). An even more significant correlation was found when Hpa2 expression was retrieved from a large number (*n* = 603) of ER-positive, HER2-negative breast cancer patients subjected to analysis by publically available software (KM plotter service; https://kmplot.com/analysis). In this cohort, the overall survival of patients exhibiting high levels of Hpa2 was considerably shorter than Hpa2-low patients [198.44 vs. 99 months for Hpa2-low vs. Hpa2-high patients, respectively; HR = 1.59 (1.1–2.3)], differences that are statistically highly significant (*p* = 0.01; Fig. 1C). In addition, we employed a tissue array of 150 invasive ductal carcinoma biopsies (median age 49; 33–75; Supplementary Table 2) and correlated the staining intensity of Hpa2 (Supplementary Fig. 1A) with molecular parameters. Notably, strong staining of Hpa2 was associated with a high rate of cell proliferation (Ki67; *p* = 0.01; Table 2) and with HER2 expression levels (Table 2; *p* = 0.06), parameters that are closely associated with disease progression. We have reported previously that the expression of heparanase in the metastatic lesion does not always reflect its expression in the primary tumor [25], discordance that may compromise the efficacy of anti-heparanase medication. We next followed this approach and subjected 42 pairs of biopsies derived from the primary breast tumor and the resulting metastases to immunostaining of Hpa2. Discordance was similarly observed for Hpa2, and some of the metastases either lost (Supplementary Fig. 1B, middle panels) or gained (Supplementary Fig. 1B, lower panels) Hpa2 expression compared with the primary lesion. Importantly, patients who showed strong staining of Hpa2 in the primary lesion and the resulting metastases (Supplementary Fig. 1B, upper panels) exhibited the lowest survival time (Supplementary Fig. 1C; *p* = 0.03). Altogether, the clinical results suggest that in breast cancer, Hpa2 promotes, rather than inhibits, tumor progression.

**Table 1:** Hpa2 staining intensity correlates inversely with tumor grade and predicts a higher incidence of lymph node metastasis.

Grade	Hpa2			Total
	0	1	2	
**2** (Mod)	5 (33)	6 (43)	17 (89)	28
**3** (Poor)	10 (67)	8 (57)	2 (11)	20
*p* = 0.002	15	14	19	48^a^
**N-stage**	**Hpa2**			
	**0**	**1**	**2**	
No	12 (33)	7 (43)	4 (21)	23
Yes	5 (67)	6 (57)	15 (79)	26
*p* = 0.002	17	13	19	49^b^

^a^Not specified in 3 samples.

^b^Not specified in 2 samples.

**Table 2:** Hpa2 levels correlate with an increased number of Ki67-positive cells and HER2 levels.

Ki67	Hpa2		Total
	Weak (0–1) (%)	Strong (2) (%)	
Low (0–1)	36 (43)	48 (57)	84
High (2 + 3)	14 (23)	48 (**77**)	62
*p* = 0.01	50	96	146^a^
**HER2**			
Negative	24 (44)	30 (56)	54
Positive	26 (28)	66 (**72**)	92
*p* = 0.06	50	96	146^a^

Ki67 scoring: <1% = 0; 1–10% = 1;11–50% = 2; >50% = 3.

^a^Data of 4 patients was missing.

Bold values emphasize parameters that were mostly affected.

### Nuclear Hpa2 attenuates breast tumor growth

Careful examination of the immunostaining revealed that in normal human breast tissue, away from the tumor lesion, Hpa2 is expressed at high levels, localizing predominantly to the cell nuclei (Fig. 1D, left panels). In breast tumors, nonetheless, Hpa2 levels were not only markedly decreased but also lost their nuclear localization and appeared diffused in the cell cytoplasm (Fig. 1D, upper right). We next scored the tumor biopsies not only for the staining intensity but also for the cellular localization of Hpa2. Notably, patients in which nuclear localization of Hpa2 is partially retained (22/143; Table 3, c + N; Fig. 1D, lower right panel) exhibit no lymph node metastasis (Table 3; *p* = 0.008), suggesting that nuclear localization of Hpa2 plays a protective role in breast cancer. Additionally, nuclear localization of Hpa2 correlated with the levels of hormone receptors (ER, PR, AR; Table 3), in agreement with the notion that hormone receptor-positive cancers grow more slowly than those that are hormone receptor-negative [26, 27].

**Table 3:** Nuclear localization of Hpa2 correlates with decreased lymph node metastasis and the levels of hormones.

	Hpa2 localization		
	c + N (%)	C (%)	Total
*N-stage*			
No	22 (20)	92 (80)	114
Yes	0 (**0**)	29 (100)	29
*p* = 0.008	22	121	143^a^
*AR*			
Weak (0–1)	3 (4)	66 (96)	69
Strong (2 + 3)	19 (**26**)	55 (74)	74
*p* < 0.0001	22	121	143^a^
*ER*			
Weak (0 + 1)	6 (7)	78 (94)	84
Strong (2 + 3)	16 (**27**)	43 (73)	59
*p* = 0.002	22	121	143^a^
*PR*			
Weak (0–1)	9 (9)	92 (91)	101
Strong (2 + 3)	13 (**31**)	29 (69)	42
*p* = 0.002	22	121	143^a^

Receptor scoring: 0 < 1%; 1 = 1–10%; 2 = 11–50%; 3 > 50%.

^a^Information of 7 patients is missing.

Bold values emphasize parameters that were mostly affected.

**Fig. 2:**
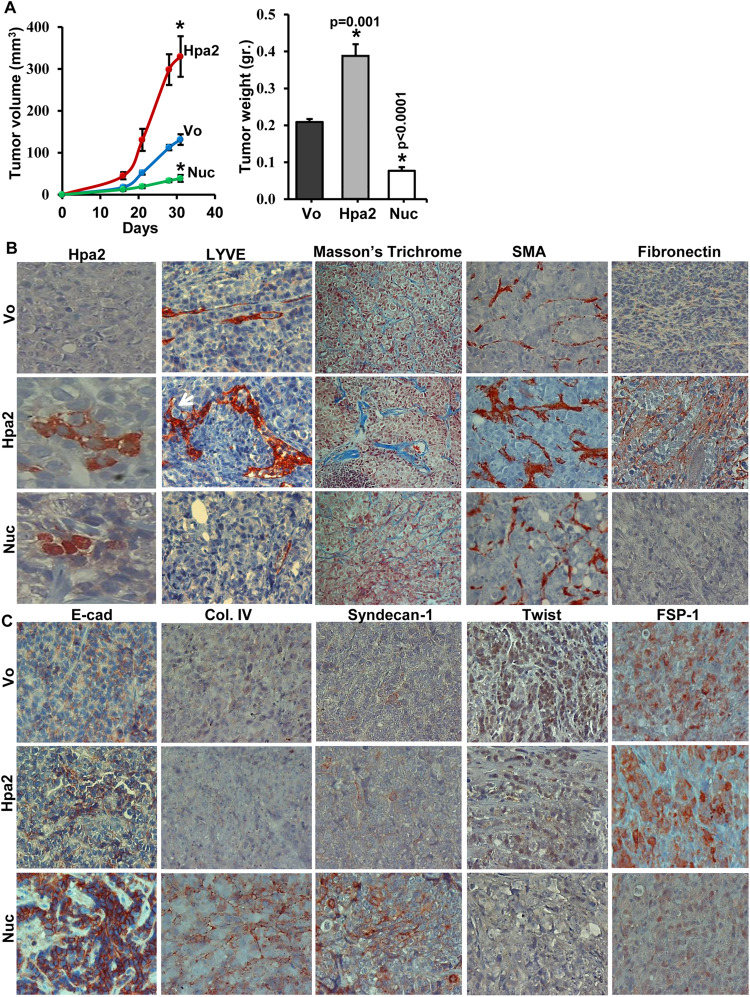
Hpa2 promotes, while nuclear Hpa2 (Hpa2-Nuc) attenuates tumor growth: ZR-75-1 cell model. **A** Tumor growth. ER-positive ZR-75-1 cells were transfected with control, empty vector (Vo), Hpa2, or Hpa2-Nuc vectors. Following selection, cells (5 × 10^6^/50 µl) were implanted orthotopically into the mammary gland of NOD/SCID mice (*n* = 7 per group), and tumor growth was inspected by caliper measurements (**A**, left). Upon termination, tumors were harvested and weighed (**A**, right). **B** Immunostaining. Five-micron sections of the indicated tumors were subjected to immunostaining applying anti-Hpa2 (left panels), anti-LYVE (a marker for lymphatic endothelium; second left), anti-SMA (second right), and anti-fibronectin (right panels) antibodies. Masson’s Trichrome staining is shown in the middle panels. **C** EMT markers. Five-micron sections of the indicated tumors were subjected to immunostaining applying antibodies directed against E-cadherin (E-Cad; left panels), collagen IV (Col. IV; second left), syndecan-1 (middle), Twist (second right) and FSP1/S100A4 (right panels). Shown are representative images at original magnifications of ×100.

In order to further examine the role of nuclear Hpa2 in breast cancer, we engineered a Hpa2 gene construct in which the signal peptide of Hpa2 (amino acids 1–38; see “Materials and methods” section) was removed, and a nuclear localization sequence (NLS) was introduced at the protein C-terminus (Hpa2-Nuc; Supplementary Fig. 1D, upper panel). Following validation that Hpa2-Nuc is indeed directed to the cell nucleus (Supplementary Fig. 1D, lower panels), we transfected breast carcinoma cells with control, empty vector (Vo), Hpa2, or Hpa2-Nuc gene constructs (Supplementary Fig. 1E) and examined their behavior. Employing the Boyden chamber and a more quantitative IncuCyte technology [24], we found that over-expression of Hpa2 stimulates the invasion and migration of ZR-75-1 and MCF10Ca cells vs. control (Vo) or Hpa2-Nuc cells (Supplementary Fig. 2A), and this increase in motility was abrogated by heparin or anti-Hpa2 monoclonal antibody (1c7) that targets a presumed HS-binding domain of Hpa2 [22] (Supplementary Fig. 2B), suggesting that the increase in cell motility is mediated by the interaction of Hpa2 with HS. We next implanted the cells orthotopically in the mammary gland of SCID mice and tumor growth was inspected. Notably, ZR-75-1 cells over-expressing Hpa2 developed tumors that were 2-fold bigger than tumors developed by control (Vo) cells in volume (Fig. 2A, left panel; *p* < 0.001) and weight (Fig. 2A, right panel; 0.21 ± 0.008 vs. 0.39 ± 0.03 g for Vo vs. Hpa2, respectively; *p* = 0.0002). In striking contrast, tumors developed by ZR-75-1 cells expressing Hpa2-Nuc developed tumors that were 2.5-fold smaller than control (Fig. 2A; 0.21 ± 0.008 vs. 0.008 ± 0.001 g for Vo vs. Hpa2-Nuc, respectively; *p* < 0.001), implying that Hpa2 promotes, while nuclear localization of Hpa2 (Fig. 2B, left panels) restrains tumor growth. Likewise, tumors developed by ZR-75-1 cells over-expressing Hpa2 exhibited an increased density of lymph vessels, whereas a lower number of lymphatic vessels was found in tumors developed by ZR-75-1 cells expressing Hpa2-Nuc (Fig. 2B, second left). Moreover, Hpa2 tumors appeared more fibrotic, evidenced by Masson’s Trichrome, SMA, and fibronectin staining (Fig. 2B, middle, second right and right panels), determinants that are associated with disease progression and tumor metastasis [28**–**30]. In contrast, the expression of E-cadherin was increased markedly in Hpa2-Nuc tumors (Fig. 2C, left panels). Moreover, Hpa2-Nuc induces the deposition of collagen IV, and the cells seem to assemble a basement membrane typical of normal epithelium that is practically absent from control (Vo) and Hap2 tumors (Fig. 2C, second left). This, the induction of syndecan-1 and decreased levels of Twist (Fig. 2C, second right), and FSP1/S100A4 (right panels) suggest that nuclear Hpa2 functions to reduce epithelial-to-mesenchymal transition (EMT) [31] and maintain the characteristics of breast epithelial cells.

**Fig. 3:**
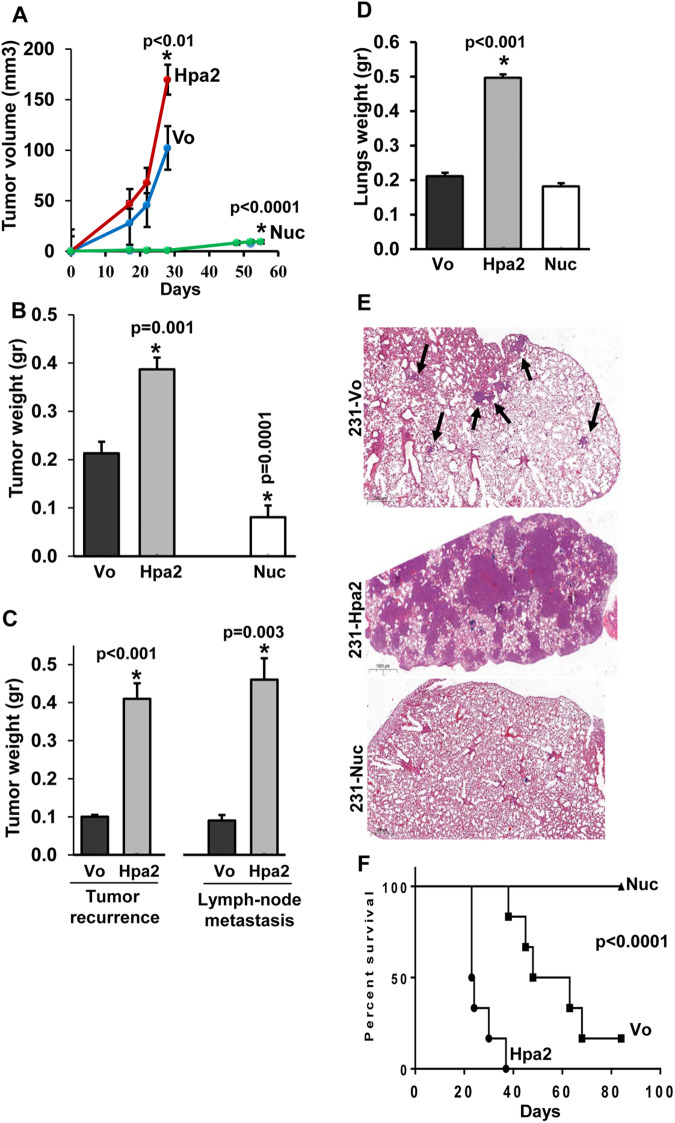
Hpa2 promotes, while nuclear Hpa2 (Hpa2-Nuc) attenuates tumor growth: MDA-MB-231 cell model. Triple-negative MDA-MB-231 cells were transfected with control, empty vector (Vo), Hpa2, or Hpa2-Nuc vectors. Following selection, cells (2.5 × 10^6^/50 µl) were implanted orthotopically into the mammary gland of NOD/SCID mice (*n* = 7 per group), and primary tumor growth was inspected by caliper measurements (**A**). After 4 weeks, when tumors reached the size of ~7 × 7 mm (~170 mm^3^), tumor xenografts were removed under anesthesia and weighed (**B**). By that time, no measurable tumors were detected in mice implanted with Hpa2-Nuc cells. Wounds were sutured, and mice were kept for an additional 4 weeks to enable metastases to grow. Mice were then sacrificed, and recurred tumors, swollen sentinel lymph nodes (**C**), and lungs (**D**) were collected and weighed. By that time, mice implanted with Hpa2-Nuc cells developed small tumors that were collected and weighed (**B**, Nuc). **E** Lung metastasis. 5-micron sections of formalin-fixed, paraffin-embedded lungs were subjected to H&E staining to visualize lung metastasis. Shown are representative lung images at ×5 magnification. Note that lung metastasis is promoted prominently by Hpa2, while nuclear targeting of Hpa2 prevents lung metastasis (Nuc). **F** Survival analyses. Control (Vo), Hpa2, and Hpa2-Nuc MDA-MB-231 cells (2.5 × 10^6^/50 µl) were implanted orthotopically into the mammary gland of NOD/SCID mice (*n* = 7 per group), and survival of the mice was recorded. Shown is a KM survival graph recorded for 90 days. Note that the survival of the mice is prolonged prominently by targeting Hpa2 to the cell nucleus.

In order to further confirm these results and examine the role of Hpa2 in tumor metastasis, we next employed MDA-MB-231 breast carcinoma cells. Applying in vitro systems, we observed that over-expression of Hpa2 in these cells (Supplementary Fig. 3A) promoted cellular invasion through re-constituted ECM (Matrigel) and colony formation in soft agar (Supplementary Fig. 3B, right panels; Supplementary Fig. 3C). In contrast, cell migration, cell invasion, cell proliferation and colony formation in soft agar were attenuated markedly by MDA-MB-231 cells over-expressing Hpa2-Nuc (Supplementary Fig. 3B–D). Importantly, overexpression of Hpa2 resulted in 2-fold bigger tumors (Fig. 3A; 0.21 ± 0.02 vs. 0.39 ± 0.02 gr for Vo and Hpa2 cells, respectively; *p* = 0.001). In striking contrast, no measurable tumors were developed by MDA-MB-231-Nuc cells after 4 weeks, when the primary tumors were resected (Fig. 3A). Four weeks thereafter, mice were sacrificed, and tumor recurrence and lymph nodes and lung metastases were examined. By that time, MDA-MB-231-Nuc cells developed tumor xenografts that were 2.3-fold smaller than tumor xenografts produced by control (Vo) cells (that were removed 4 weeks earlier) (0.09 ± 0.01 gr; Fig. 3B, Nuc). Tumor recurrence was noted in all mice implanted with control (Vo) and MDA-MB-231-Hpa2 cells (Supplementary Fig. 4, red arrows). Remarkably, recurred MDA-MB-231-Hpa2 tumors were 4-fold bigger than Vo tumors (0.1 vs. 0.41 gr for Vo and Hpa2 tumors, respectively; *p* < 0.001; Fig. 3C). Moreover, metastasis to sentinel lymph nodes was found in 3/7 mice implanted with control (Vo) cells, and in all mice (7/7) implanted with Hpa2 cells (Supplementary Fig. 4A, white arrows), and these nodes were over four-fold bigger by weight (0.09 vs 0.46 for Vo and Hpa2 tumors, respectively; *p* = 0.003; Fig. 3C). Furthermore, 5/7 mice implanted with Hpa2 cells exhibited metastatic lesions also in the opposite mammary gland (Supplementary Fig. 4A, black arrows; Supplementary Fig. 4B), implying that MDA-MB-231 cells over-expressing Hpa2 develop more aggressive and more metastatic disease. Indeed, histological examination revealed massive metastasis in the lungs of mice implanted with MDA-MB-231-Hpa2 cells vs control (Vo) cells (Fig. 3E), associating with a marked increase in lymph vessel density (LYVE; Supplementary Fig. 5A, upper and second panels). Strikingly, no metastatic lesions were observed in the lungs of mice implanted with MDA-MB-231-Hpa2-Nuc cells (Fig. 3E, lower panel), thus strongly implying that targeting Hpa2 to the cell nucleus reduces their tumorigenicity and metastatic capacities substantially. This is best demonstrated by survival analyses. Remarkably, the survival of mice implanted with MDA-MB-231 cells over-expressing Hpa2 was markedly reduced vs control mice (23.5 vs. 55.5 days for mice implanted with Hpa2 and control (Vo) cells; *p* < 0.001). In striking contrast, the survival of mice implanted with MDA-MB-231-Hpa2-Nuc cells was impressively prolonged (Nuc; Fig. 3F), implying that when localized to the cell nucleus, Hpa2 restrains tumor growth and metastasis. Immunostaining further revealed that Hpa2-tumors exhibit a higher rate of cell proliferation indicated by a two-fold increase in Ki67-positive cells (Supplementary Fig. 5A, lower panels), whereas halted tumor growth by Hpa2-Nuc cells is associated with substantial recruitment of macrophages (F4/80; Supplementary Fig. 5A, third panels).

**Fig. 4:**
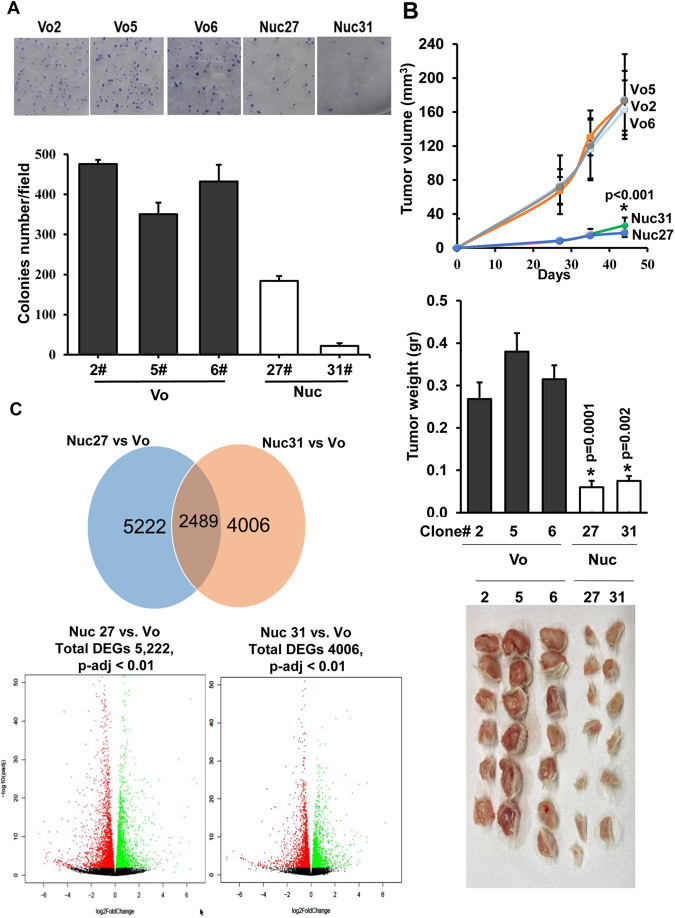
Selection of cell clones and RNAseq analysis. **A** Soft agar. ZR-75-1-Nuc cell clones were isolated and examined for high expression of Hpa2-Nuc. Two such cell clones (#27, #31) were selected and compared to cell clones that were selected randomly from control (Vo) cultures (#2, #5, #6) for their capacity to form colonies in semi-solid (soft) agar (**A**, upper panel). Quantification of colony number is shown graphically in **A**, lower panel. **B** Tumor growth. Cells (5 × 10^6^) from the indicted clone were inoculated orthotopically in the mammary gland of NOD/SCID mice (*n* = 7), and tumor volume was inspected using caliper measurements over time (upper panel). At termination, tumors were collected, weighed (middle panel), and photographed (lower panel). Portion of the tumor was excised for the isolation of RNA, and the other portion was fixed in formalin for histological analysis. **C** RNAseq analysis. Total RNA was extracted from tumors generated by control (Vo; #2, #5, #6; *n* = 6) and Hpa2-Nuc (#27, #31; *n* = 6) cell clones and subjected to RNAseq analysis. Ven diagram for altered gene expression common to Nuc #27 vs. Vo and to Nuc #31 vs. Vo is shown in the upper panel. Volcano plots of genes that were down-regulated (red) or up-regulated (green) by Nuc 27 vs. Vo cell clones (left) and Nuc 31 vs. Vo cell clones (right), using *p*-adj < 0.01, are shown in **C**, lower panels.

In order to further investigate the role of nuclear Hpa2 on tumor growth, we selected ZR-75-1 cell clones that express high levels of Hpa2-Nuc (#27, #31) (Supplementary Fig. 5B) and compared their tumorigenic capacity to cell clones that were selected randomly from control (Vo) cultures (#2, #5, #6). We found that Hpa2-Nuc cell clones developed fewer colonies in soft agar vs. control cells (Fig. 4A; *p* = 0.003 and 0.0004 for Nuc#27 vs Vo#5 and Nuc#31 vs. Vo#5, respectively), a property thought to reflect the tumorigenic potency of the cells. Importantly, Hpa2-Nuc cell clones developed tumors that were 6-fold smaller than tumors produced by control cell clones (Fig. 4B), differences that are statistically highly significant (*p* = 0.0001 and 0.002 for Nuc#27 and Nuc#31 vs. Vo#2, respectively). These results further support the notion that nuclear localization of Hpa2 attenuates tumor growth.

### Nuclear localization of Hpa2 affects the expression of genes associated, among others, with angiogenesis, interferon, and metabolism

**Fig. 5:**
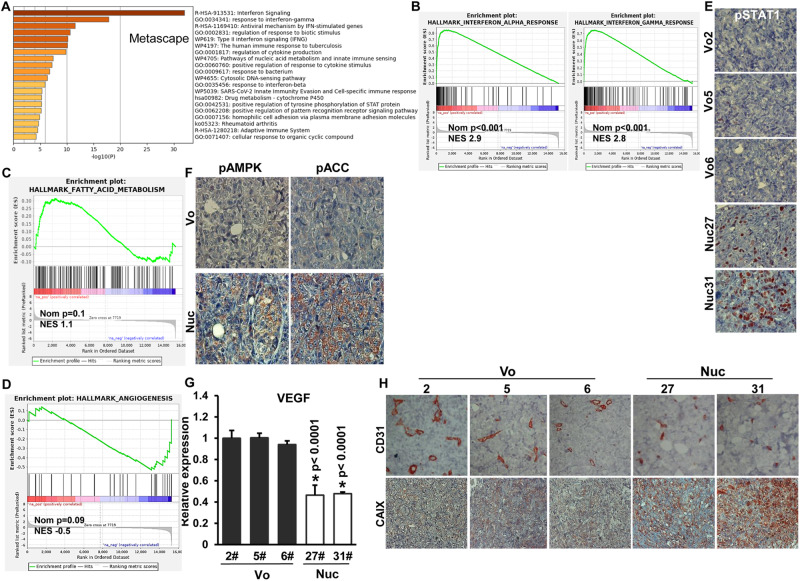
Targeting of Hpa2 to the nucleus of ZR-75-1 cells (Nuc) elicits pathways associated with interferon signaling, fatty acids metabolism, and angiogenesis. **A** and **B** Interferon pathway. **A** Pathway analysis of the DEG placed interferon signaling, response to interferon-gamma, and type II interferon signaling at the top of the list of pathways elicited by Hpa2-Nuc. **B** Gene set enriched analysis (GSEA) further pointed to the enrichment of genes associated with interferon-alpha and interferon-gamma responses. **C** Fatty acids metabolism: GSEA also pointed to the enrichment of genes associated with fatty acid metabolism. **D** Angiogenesis: GSEA pointed to decreased expression of genes associated with angiogenesis. **E** STAT1 phosphorylation: Tumor sections were subjected to immuno-staining applying anti-phospho-STAT1 antibody. Shown are representative photomicrographs at the original magnification of ×100. Note strong nuclear staining of phospho-STAT1 only in tumor xenografts produced by Hpa2-Nuc cells. **F** Immunostaining: Tumor sections produced by control (Vo) and Hpa2-Nuc cells were subjected to immuno-staining applying phopsho-AMPK (pAMPK; left panels) and phospho-ACC (pACC; right panels) antibodies. **G** qPCR analysis revealed that VEGF expression is decreased two-fold in tumors produced by Hpa2-Nuc cells vs control tumors. VEGF expression by Nuc cells is presented relative to control (Vo #2) cells, set arbitrarily to a value of 1, and calculated after normalization to actin. **H** Immunostaining: 5-micron sections of tumors produced by control (Vo) and Hpa2-Nuc cell clones were subjected to immunostaining applying anti-CD31 (PECAM; a marker of endothelial cells, upper panels) and anti-carbonic anhydrase IX (CAIX; a marker for hypoxia; second panels) antibodies. Note impaired vasculature and hypoxic tumors produced by Hpa2-Nuc cells.

To reveal the molecular mechanism associated with tumor growth inhibition by nuclear Hpa2, we extracted total RNA from tumors produced by control (#2, #5, #6) and Hpa2-Nuc (#27, #31) cell clones and employed RNAseq methodology to reveal genes regulated by nuclear Hpa2 (Hpa2-Nuc). For analysis, we selected differentially expressed genes (DEG) using *p*-adj < 0.01 for Nuc clones vs control (Vo) clones (i.e., Nuc #27 vs. Vo, Nuc #31 vs. Vo; Fig. 4C). We applied Metascape, ingenuity pathway analysis (IPA; QIAGEN) and gene set enrichment analysis (GSEA) tools to reveal the function of gene sets regulated by Hpa2-Nuc. We found that Hpa2-Nuc affects the expression of many genes regulated by interferon (Fig. 5A, B), genes involved in the metabolism of fatty acids (Fig. 5C), and genes that regulate angiogenesis (Fig. 5D). We further validated these pathways by quantifying, for example, a marked increase in interferon-induced protein 44 (IFI44), interferon-induced protein 44-like (IFI44L), and interferon-inducible protein 10 (IP-10; CXCL10) expression by Hpa2-Nuc tumors (Supplementary Fig. 6A–C). Likewise, we revealed a marked increase in STAT1 phosphorylation (Fig. 5E); increased phosphorylation of AMPK, a pathway that plays a critical role in fatty acid synthesis and fatty acid oxidation [32], and its substrate, ACC, in Hpa2-Nuc tumors (Fig. 5F), along with decreased expression of VEGF-A (Fig. 5G). We also found that the vasculature in Hpa2-Nuc tumors is disrupted and appears abnormally collapsed (i.e., lack a patent lumen; Fig. 5H, upper panels). Augmented tumor hypoxia, evident by increased staining intensity of carbonic anhydrase IX in Hpa2-Nuc tumors (CAIX; Fig. 5H, lower panels), further supports the notion that blood vessels in these tumors do not function properly. Perturbed tumor vasculature and activation of AMPK and interferons are well-established anti-tumorigenic pathways [33**–**35] that likely underline, at least in part, the attenuation of tumor growth by Hpa2-Nuc.

### Hpa2 promotes Myc signaling

**Fig. 6:**
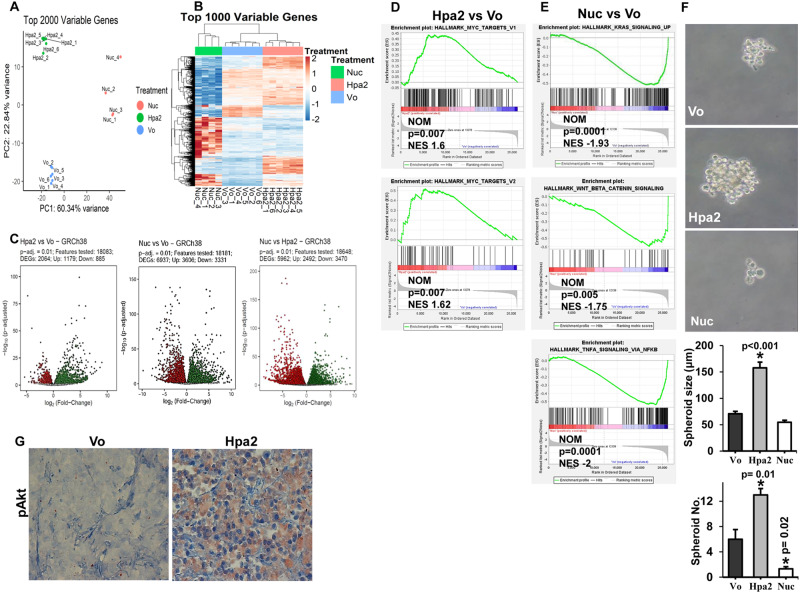
RNAseq analysis of MDA-MB-231 Vo, Hpa2, and Hpa2-Nuc tumor xenografts. Total RNA was extracted from tumor xenografts produced by control MDA-MB-231 control (Vo) cells (*n* = 6), Hpa2-over expressing cells (*n* = 6), and Hpa2-Nuc cells (*n* = 4) and subjected to RNAseq analysis. **A** PCA plot: Descriptive analysis of the samples, showing clusters of samples based on their similarity. Note that the study groups (Vo, Hpa2, Hpa2-Nuc) exhibit high similarity among the samples within each group and appear distinct from each other. **B** Heatmap description of the top 1000 variable genes. **C**. Volcano plots: *x*-axis shows the fold-change in gene expression between two groups; The *y*-axis shows the statistical significance of the differences. Dots marked in gray represent genes without significant differential expression; Dots marked in green represent significantly upregulated genes **(***p*-adj < 0.01); Dots marked in red represent significantly downregulated genes (*p*-adj < 0.01). **D** and **E** GSEA analyses: Subjecting the list of differentially-expressed genes to GSEA analysis pointed to the induction of genes related to the Myc pathway in Hpa2 vs. Vo tumors (**D**), while genes that signify the hallmark of KRas, beta-catenin, and TNF-alpha were repressed in Nuc vs. Vo tumors (**E**). **F** Spheroid assay. Control (Vo), Hpa2, and Nuc cells (3 × 10^3^) were cultured in 24-well ultralow attachment plates as described under the ‘Materials and methods’ section. Tumor spheres were photographed (upper panels), measured (fourth panel), and counted (lower panel) after 10 days in culture. **G** Immunostaining. 5-micron sections of tumor xenografts produced by control (Vo) and 231-MDA-MB-Hpa2 cells were subjected to immunostaining applying anti-phospho-Akt antibody. Shown are representative images at the original magnification of ×100.

To further examine the molecular mechanism(s) underlying the pro-tumorigenic properties of Hpa2 in breast cancer progression, we next examined DEG in the MDA-MB-231 cell model. RNAseq analysis revealed that overexpression of Hpa2 and its nuclear targeting (Hpa2-Nuc) results in a distinct pattern of gene expression (Fig. 6A, B). Volcano plots displaying many genes that are induced or repressed by Hpa2 and Hpa2-Nuc are depicted in Fig. 6C. GSEA analysis of Hpa2 transcriptome pointed to enrichment of genes induced by Myc (Fig. 6D), whereas expression of genes associated with the hallmark of Kras, beta-catenin, and TNF-alpha (via NFkB) signaling was repressed in the Hpa2-Nuc transcriptome (Fig. 6E). Myc is highly implicated in different aspects of breast cancer tumorigenesis, including a most prominent function in the establishment of cancer stem cells [36**–**38]. Applying the spheroid assay as an indication of cancer stem cells, we found that MDA-MB-231-Hpa2 cells developed bigger (Fig. 6F, fourth panel) and more (Fig. 6F, lower panel) spheroids vs spheroids produced by control (Vo) cells, whereas smaller and fewer spheroids were formed by Hpa2-Nuc cells (Fig. 6F; *p* < 0.001 and *p* = 0.01 for Vo vs. Hpa2 and Vo vs. Nuc, respectively). In addition, we found an elevation of Akt phosphorylation in tumors produced by MDA-MB-231-Hpa2 cells (Fig. 6G), a signaling pathway implicated in Myc-mediated cell survival [39].

### Nuclear targeting of Hpa2 affects the tumor microenvironment

**Fig. 7:**
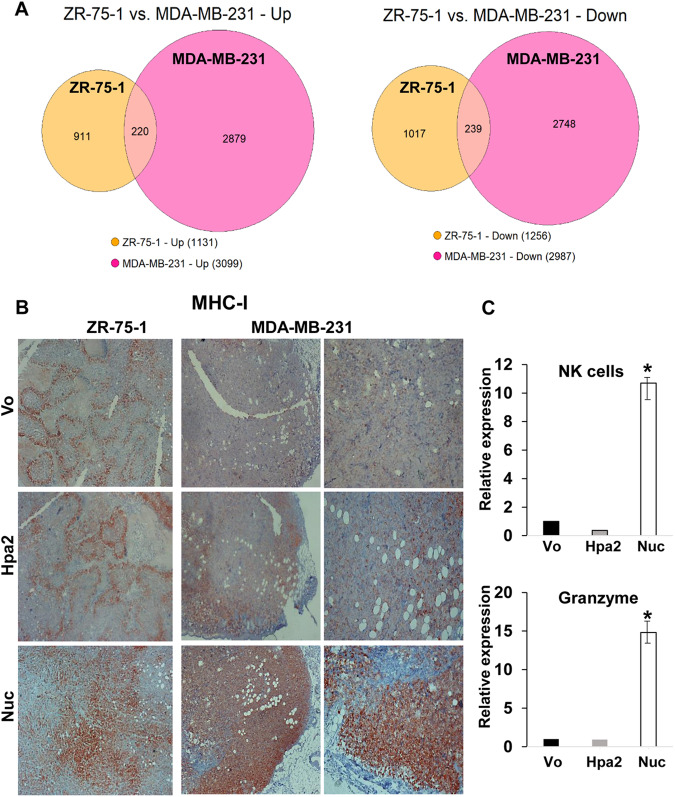
Comparison of the RNAseq profiles obtained with ZR-75-1 and MDA-MB-231 cells overexpressing Hpa2-Nuc points to alterations in the tumor microenvironment. **A** Venn diagrams: Expression of 220 genes that were induced (left panel) and 239 genes that were repressed (right panel) by Hpa2-Nuc are in common to both cell models. **B** MHC-I immunostaining: 5-micron sections of ZR-75-1 (left panels) and MDA-MB-231 (middle and right panels) tumors were subjected to immunostaining applying anti-MHC-I antibody. Shown are representative images at original magnifications of ×25 (middle panels) and x100 (left and right panels). **C** qPCR: Total RNA was extracted from ZR-75-1 control (Vo), Hpa2, and Hpa2-Nuc tumors and subjected to qPCR analysis applying primer sets specific for NK cells (NK-1.1; upper panel) and granzyme (lower panel). Gene expression by Hpa2/Nuc tumors is presented relative to tumors produced by control (Vo) cells, set arbitrarily to a value of 1, and calculated after normalization to actin.

To reveal genes affected by nuclear targeting of Hpa2 in both ZR-75-1 (ER-positive) and MDA-MB-231 (triple-negative) cell models, we compared their RNAseq profiles. We found that 220 genes that were induced and 239 genes that were repressed by nuclear targeting of Hpa2 were in common in both cell models (Fig. 7A). Notably, genes that are associated with the tumor microenvironment emerged as most affected by Hpa2-Nuc cells. Among others, these included induction of interferon-inducible genes [i.e., interferon regulatory factor 5 (IRF5), interferon-induced transmembrane protein 3 (IFITM3), interferon-induced protein 35 (IFI35)] and cytokines (i.e., CXCL14, CCL5). Interestingly, CXCL14 is considered critical to the upregulation of the major histocompatibility complex (MHC) class I expression in tumor cells [40]. Indeed, we found a marked increase in MHC-I immunostaining in tumors produced by ZR-75-1-Nuc and MDA-MB-231-Nuc cells (Fig. 7B), along with a substantial increase in HLA-F (class I), HLA-DRB1 and HLA-DRB5 (class II) gene expression in Hpa2-Nuc tumors. Moreover, we found an over 10-fold increase in the number of NK cells recruited to Hpa2-Nuc tumors and an over 15-fold increase in the level of granzyme in these tumors (Fig. 7C). More efficient presentation of tumor antigens in the context of MHC molecules, and efficient recruitment of immune cells, likely underline the better outcome of breast cancer patients that retain nuclear localization of Hpa2.

## Discussion

In women, breast cancer is the most commonly diagnosed cancer and the leading cause of cancer death [41]. While major advances have been made toward breast cancer prevention and treatment, the incidence of breast cancer is still increasing globally, encouraging the development of new technologies for early and more accurate diagnosis [42]. In addition, a deeper understanding of the molecular biology and immunology aspects of the disease is required to improve the diagnosis and develop highly targeted therapies. Here, we examined the expression and clinical significance of Hpa2 in cohorts of women diagnosed with breast cancer. Unexpectedly, we found that breast cancer patients exhibiting strong staining intensity of Hpa2 survived less than patients showing low levels of Hpa2. The association between high levels of Hpa2 and poor prognosis of breast cancer patients stands in striking contrast with previous reports, showing that high levels of Hpa2 are associated with prolonged survival of cancer patients [2, 16, 19, 20, 43, 44]. The unexpected, pro-tumorigenic role of Hpa2 in breast cancer seems to involve its cellular localization. This notion emerges from the observation that Hpa2 resides primarily in the nucleus of normal breast epithelium but loses its nuclear residence and appears markedly decreased and diffused in the cytoplasm of cancer cells. In breast malignancy, the secreted Hpa2 appears to promote, while nuclear Hpa2 attenuates tumorigenesis.

### Hpa2

Association between high levels of Hpa2 and poor prognosis was found in three cohorts of breast cancer patients. The first cohort included 51 young patients in which the disease is thought to be more aggressive. Shorter survival of patients exhibiting high levels of Hpa2 was associated with increased tumor metastasis to regional lymph nodes (N-stage); The second cohort included primary breast tumors and their resulting metastases. We found discordance in Hpa2 expression, and the primary lesion gained or lost its expression in 54% (19/35) of the cases. Mechanisms that induce the expression of Hpa2 are only starting to emerge and mainly focus on conditions of stress [16, 19, 20]. However, the mechanism(s) underlying a decrease in Hpa2 expression in breast carcinoma vs normal breast epithelium (Fig. 1) [13] or in the primary lesion vs. the resulting metastasis (Supplementary Fig. 1B) is currently unclear. Noteworthy, hypermethylation of the Hpa2 promoter was reported to down-regulate Hpa2 expression, and the resulting low levels of Hpa2 were associated with shorter survival of colorectal cancer patients [43]. A similar epigenetic mechanism may also occur in breast carcinoma, but this possibility needs to be approved. Importantly, patients that exhibited strong staining of Hpa2 in the primary lesion and its metastases (P^+^M^+^) survived significantly less than patients in any of the other groups (i.e., P^−^M^−^, P^+^M^−^, P^−^M^+^). Further analysis utilizing a publically available software (KM plotter service; https://kmplot.com/analysis) revealed a shorter survival of patients with Hpa2-high tumors in a large cohort of over 600 ER-positive, HER2-negative breast cancer patients (Fig. 1). In addition, we examined a cohort of 150 breast cancer patients diagnosed at older age. Unfortunately, we did not have the survival data of these patients. However, strong staining intensities of Hpa2 were associated with a high rate of cell proliferation (i.e., Ki67-positive cells) and high levels of HER2, which play a key role in the progression of breast cancer. Altogether, the clinical evidence strongly directs toward pro-tumorigenic properties of Hpa2 in breast cancer. Importantly, the clinical data were closely recapitulated by two preclinical tumor models. Given the low levels of endogenous Hpa2 in the tumor-derived breast carcinoma cell lines (Supplementary Fig. 6E), silencing of Hpa2 was not feasible. Over-expression of Hpa2 in ER-positive ZR-75-1 and triple-negative MDA-MB-231 cells resulted in bigger and more aggressive tumors. This is best exemplified by the MDA-MB-231 cell model, where over-expression of Hpa2 promoted tumor growth and lung metastasis, resulting in a marked decrease in the survival of the mice (Fig. 3). Moreover, tumors developed by MDA-MB-231-Hpa2 cells exhibited far greater number of lymph vessels, many of which were densely infiltrated with tumor cells (Supplementary Fig. 5A). This result support the notion that Hpa2 is intimately engaged in tumor vascularity [22, 24].

RNAseq methodology revealed that Hpa2 apparently regulates the expression of many genes, and the GSEA tool revealed the hallmark of Myc target genes to be primarily affected by Hpa2. The protooncogene Myc is situated downstream of many signaling pathways; Myc is highly associated with proliferation, differentiation, apoptosis, and self-renewal in various types of cancer, including breast cancer [45**–**47]. Indeed, we were able to confirm that Hpa2 tumors exhibit a higher rate of cell proliferation and increased levels of Akt phosphorylation, a key kinase downstream of the PI-3K signaling pathway implicated in Myc signaling [39]. In addition, Hpa2 cells form more and bigger spheroids which reflect the occurrence of cancer stem cells, a most typical feature of Myc function [38].

The mechanism(s) by which Hpa2 affects gene transcription and tumor growth and metastasis is not entirely clear but may involve HSPG. This possibility emerges from the capacity of heparin and anti-Hpa2 monoclonal antibody 1c7, which target a presumed HS-binding domain [22] to inhibit the pro-migratory capacity of Hpa2 (Supplementary Fig. 2). Given the secreted nature of Hpa2 [2], the prime suspects that modulates cell motility are cell membrane HSPG such as syndecans and glypicans. For example, it was reported recently that high levels of glypicans are associated with shorter survival of HER2-negative and triple-negative breast cancer patients [48], suggesting that Hpa2 may affect the disease via HSPG. This possibility awaits thorough investigation.

### Nuclear Hpa2

In striking contrast with the secreted protein, breast tumors that retain nuclear Hpa2 exhibited no lymph node metastasis (Table 3). Furthermore, targeting Hpa2 to the nucleus of breast carcinoma cells prominently decreased their tumorigenic capacities, best exemplified by decreased lung metastasis and prolonged survival of mice implanted with MDA-MB-231-Hpa2-Nuc cells. Furthermore, MDA-MB-231-Hpa2-Nuc cells were far more sensitive to conditions of anoikis (i.e., lack of cell attachment) [49], resulting in over 3-fold increase in cell death vs control (Vo) and Hpa2 cells, and exhibited decreased number and size of cancer stem-cells spheroids (Fig. 6F and Supplementary Fig. 6D). These results, the strong induction of E-cadherin, collagen IV, and syndecan-1 (Fig. 2) and the marked decrease in tumor lymph-angiogenesis underlie, at least in part, the strong anti-tumorigenic and anti-metastatic function of nuclear Hpa2. Importantly, targeting Hpa2 to the cell nucleus is based on the observation that it localizes predominantly in the nuclei of normal breast epithelial cells, implying that nuclear targeting of Hpa2 is physiologically relevant.

Pathways responsible for the nuclear translocation of Hpa2 are presently unclear. It should be noted nonetheless that several HSPGs were also found to assume nuclear localization, possibly attenuating tumor spread [50]. Thus, Hpa2 can reach the cell nucleus while in complex with HSPG due to the high-affinity interaction between Hpa2 and HSPG [2]. The importance of HS side chains in this function emerged from studies showing that by cleaving HS side chains, heparanase enhances histone acetyltransferase activity and elicits a more aggressive tumor phenotype [50**–**52]. The ability of Hpa2 to inhibit the HS-cleaving activity of heparanase [2, 53] may take place also in the nucleus, thus tilting the heparanase:Hpa2 ratio in favor of Hpa2. In addition, Hpa2 likely interacts with nuclear HSPG, a high-affinity interaction that can lead to alterations in chromatin structure and modification in gene transcription. Our results clearly indicate that targeting Hpa2 to the cell nucleus affects the expression of a gene set distinct from the one regulated by the secreted Hpa2 (Fig. 6A, B). Among others, nuclear Hpa2 elicited the interferon response, a well-established anti-tumor pathway [54], best exemplified by a prominent increase of STAT1 phosphorylation in Hpa2-Nuc tumors (Fig. 5E). In addition, Hpa2-Nuc resulted in decreased tumor angiogenesis and tumor fibrosis (Supplementary Fig. 6F), two well-established constituents of the tumor-supporting microenvironment. In fact, examining the gene sets affected by Hpa2-Nuc in both the ZR-75-1 and MDA-MB-231 cell models pointed to pathways that relate to the tumor microenvironment (Fig. 7), including increased levels of MHC-I, responsible for the presentation of tumor antigens to cells of the immune system and noticeable recruitment of NK cells to Hpa2-Nuc tumors (Fig. 7). Thus, while decreased KRas, beta-catenin, and TNFα signaling likely underly the attenuation of tumor growth by Hpa2-Nuc in pre-clinical models (Fig. 6), activation of the immune system likely play a suppressive role in breast cancer patients in which nuclear localization of Hpa2 was retained (Fig. 1D). This novel finding may be exploited clinically by therapeutics (i.e., Selinexor) aimed to attenuate the export of nuclear tumor suppressors.

## Materials and methods

### Study population

Paraffin blocks were obtained from 61 young patients (ages 19–45) diagnosed with breast cancer and treated at the Rambam Health Care Campus, Haifa, Israel, between the years 1990 and 2014. All patients received standard-of-care treatment for breast cancer, according to the time of diagnosis, and were under surveillance in the Oncology Department, Rambam Health Care Campus, Haifa, Israel. Their performance was analyzed in correlation with pathological, demographic, and clinical characteristics, including stage of disease (TNM), pathological grade, estrogen and progesterone receptor status (ER, PR), HER2 expression (where available), metastatic disease, and treatment modality (chemotherapy, trastuzumab, hormonal and radiation therapies). Patients were excluded from final analyses if tissue samples were not available for staining. The study was approved by the hospital’s Helsinki Committee. Biopsies were subjected to immunostaining, applying anti-Hpa2 antibody essentially as described [2, 16, 19], and the staining was scored according to the intensity (0: none; +1: weak-moderate; +2: strong) in the malignant cells by an expert senior pathologist in a blinded manner. Specimens that were similarly stained with normal rabbit serum or by applying the above procedure but lacking the primary antibody yielded no detectable staining. Similarly, we subjected biopsies of the primary breast tumor and the resulting metastases to immunostaining of Hpa2 (*n* = 42), and the intensity of staining was correlated with the clinical records. A tissue array of 150 breast tumors was also subjected to immunostaining for Hpa2, and the intensity of the staining was analyzed in correlation with the clinical records.

### Gene constructs and transfection

We first sequenced the secreted Hpa2 recombinant protein and found that serine^39^ is the first N-terminal amino acid (**S**^**39**^QAGDR), ruling out the prediction that Hpa2 is a transmembrane protein [1]. Amino acids 1–38 that constitute the protein signal peptide were removed when cloned to the pEYFP vector (Addgene; Watertown, MA, USA) that contains three nuclear localization signals (NLS) in tandem to yield the Hpa2-Nuc gene construct. Control, empty pcDNA3 vector (Vo), and vector carrying the full-length Hpa2 cDNA (Hpa2c) have been described previously [2]. Cells were transfected with the above gene constructs, selected with neomycin (G418), expanded, and pooled. Cell clones were isolated by limiting dilution and clones exhibiting high Hpa2-Nuc expression were evaluated by qPCR, carried out essentially as described [22].

### Cells, cell culture, and tumorigenicity

ER-positive ZR-75-1 human breast carcinoma cells were purchased from the Americal Type Culture Collection (ATCC; Manassas, VI, USA); MCF10Ca and triple-negative MDA-MB-231 cells were described previously [55]. The cell lines were authenticated in May 2021 by the short tandem repeat (STR) profile of 15 loci plus amelogenin for sex determination (X or XY) method, according to the manufacturer’s (Promega, Madison, WI, USA) instructions, essentially as described [56]. Cells were grown in Dulbecco’s modified Eagle’s medium (Biological Industries, Beit Haemek, Israel) supplemented with 10% FCS and antibiotics. Cell migration, cell invasion, and colony formation assays were performed essentially as described [24]. The sphere-forming capacity of cancer stem cells was performed as described previously [24]. Briefly, single-cell suspensions of MDA-MB-231 cells were cultured at 5000 cells/ml in six-well ultralow attachment plates (Corning) using serum-free DMEM/F12 medium supplemented with 20 ng/ml basic FGF, 20 ng/ml EGF, and B27 supplement (Gibco; Waltham, MA, USA). Tumor spheres were counted and photographed after 7–10 days of culture. In Anoikis assay, cells (1 × 10^6^) were cultured on Poly-HEMA-coated dishes in DMEM medium supplemented with 2.5% FCS. Cell viability was quantified after 4 days using an Annexin V-FITC apoptosis detection kit (BioLegend; San Diego, CA, USA). For xenotransplantation, cells from exponential cultures were detached with trypsin/EDTA, washed with PBS, and cell suspension (2.5–5 × 10^6^/0.05 ml) was inoculated into the third mammary fat pad of 8-week-old female NOD/SCID mice. Xenograft size was determined by externally measuring tumors in 2 dimensions using a caliper. Tumor lesions were removed after 4 weeks, weighed, fixed in formalin, and subjected to histological and immunohistochemical analyses essentially as described [19, 22, 23]. In the tumor metastasis model, mice were euthanized when tumor reached the size of ~7 × 7 mm (~180 mm^3^), typically 4 weeks after cell inoculation. The primary lesions were then removed, wounds were sutured, and the mice were kept for an additional 4 weeks. Mice were then sacrificed, and lungs were collected, fixed in formalin, and embedded in paraffin. Five-micron sections were subjected to hematoxylin and eosin (H&E) staining, and the number of metastatic lesions was evaluated.

### Antibodies and reagents

Anti-Hpa2 monoclonal (20c5, 1c7) and polyclonal (#58) antibodies have been described previously [2, 22, 57]. Anti-phospho AMPK, anti-phospho-acetyl-CoA carboxylase (ACC), anti-phospho-STAT1, anti-phospho-Akt, and anti-major histocompatibility complex class I (MHC-I) antibodies were purchased from Cell Signaling (Boston, MA, USA). Anti-LYVE, anti-carbonic anhydrase 9 (CAIX), anti-Ki67, anti-collagen IV, anti-fibronectin, anti-Twist, anti-FSP1/S100A4, and anti-E-cadherin antibodies were purchased from Abcam (Cambridge, UK); Anti-syndecan-1 antibody was purchased from Santa Cruz Biotechnology (Santa Cruz, CA, USA); Anti-CD31 (PECAM) was purchased from Dianova (Hamburg, Germany). Rat anti-mouse F4/80 antibody was purchased from Serotec (Hercules, CA, USA). Anti-alpha-smooth-muscle actin (αSMA) antibody and poly(2-hydroxyethyl methacrylate) (poly-HEMA) were purchased from Sigma (St. Louis, MO, USA). Annexin V-FITC apoptosis detection kit was purchased from R&D systems (Minneapolis, MN, USA). Breast carcinoma tissue array (BR1505b) was purchased from US Biomax (Rockville, MD, USA).

### Histology and immunohistochemistry

Paraffin-embedded 5 µm sections were subjected to H&E and Masson’s trichrome staining or were immunostained with the indicated antibody using the Envision kit, according to the manufacturer’s (Dako; Santa Clara, CA, USA) instructions, as described previously [19, 22, 23]. Slides were boiled in Universal HIER antigen retrieval reagent (Abcam) for 15 min in a pressure cooker prior to immunostaining.

### Real time-PCR

Real-time-PCR analyses were performed using ABI PRISM 7000 Sequence Detection System employing SYBR Green PCR Master Mix (Applied Biosystems, Foster City, CA, USA), essentially as described [19, 20, 22]. Gene expression in Hpa2/Nuc cells is presented as mean level (±SE) relative to control (Vo) cells, set arbitrarily to a value of 1 and calculated after normalization to actin. The primer sets utilized in this study are summarized in Supplementary Table 3.

### RNAseq analysis

All RNA samples for gene expression analysis had an RNA Integrity Number (RIN) value above 7.5 using the Experion system (Biorad; Hercules, CA, USA). Gene expression profiling was performed in the Genomics Core Facility of the Rappaport Faculty of Medicine, Technion. RNAseq libraries were constructed simultaneously using the NEBNext Ultra II Directional RNA Library Prep Kit for Illumina, according to the manufacturer’s (NEB, cat. no. E7760) protocol. For bioinformatics analysis, single reads (100 bps) were aligned to the human and mouse (GRCh38 and mm10) combined reference (refdata-gex-GRCh38-and-mm10–2020-A) using STAR (V2.5.3a). The number of reads per gene was evaluated using HTSeq-count (v2.0.2). Normalization and differential expression analyses were conducted using the DESeq2 R package (v1.36.0). The similarity between samples was evaluated within the DESeq2 package using the Euclidean distance matrix and a principal component analysis (PCA). Gene set enrichment analysis (GSEA) was performed using the GSEA graphical user interface for Windows (v4.3.2). Organism-specific (human genes) normalized count tables derived from DESeq2 analysis were used as input. The analysis was performed with default software settings except for permutation type, which was set to “gene_set” according to the guideline recommendation for a small sample size. The list of genes was ranked using the default ranking metric “Signal-2-Noise,” and gene set enrichment was tested for every gene set in the human hallmark collection (MSigDB). *p*-adj = 0.01 was applied in the analyses of DEG.

### Statistical analysis

Statistical analysis was performed using SPSS 11.5 software. The Kruskal–Wallis test was applied to define differences in Hpa2 staining intensity between the groups included in the study. Fisher exact test and Mann–Whitney test were performed to check the above-mentioned differences between the pair of groups, and Spearman’s correlation was employed to define the correlation between disease severity and the intensity of Hpa2 staining. Experimental data are presented as means ± SE, and statistical significance was analyzed by a two-tailed Student’s *t*-test. The level of significance selected to examine the various parameters in this study was set at *p* ≤ 0.05.

All preclinical animal studies were performed in compliance with the regulations and ethical guidelines for experimental animal studies, in accordance with the Technion’s Institutional Animal Care and Use Committee (IL-078-05-21; OPRR-A5026-01).

### Supplementary information

Suppl. Materials

## Data Availability

All data generated and analyzed in this study are presented in this published article and its supplementary information files. All primary data can be made available from the corresponding authors upon request.
