# Supplementary material for: Nuclear localization of heparanase 2 (Hpa2) attenuates breast carcinoma growth and metastasis
Source: Cell Death Dis. 2024 Mar 22;15(3):232. doi: 10.1038/s41419-024-06596-8 (PMC10959965; doi:10.1038/s41419-024-06596-8)
Supplement: Supplementary file 1 — Suppl. Materials [file 41419_2024_6596_MOESM1_ESM.pdf]

Suppl. Figure legends

Suppl. Figure 1. Clinical significance of Hpa2 in breast cancer. **A**. Immunostaining. Breast cancer tissue array (150 tumor samples) was subjected to immunostaining applying anti-Hpa2 antibody (#58). Shown are photomicrographs of the entire array (left) and representative tumors stained negative (o; upper right), or exhibiting weak (+1; middle) or strong (+2; lower) staining intensity. **B**. Discordance between Hpa2 staining intensity of the primary lesion and the resulting metastasis. Pairs of primary breast tumors and their resulting metastatic lesions were subjected to immunostaining applying anti-Hpa2 antibody. Shown are representative images of metastases that lost (middle panels; n=10) or gained (lower panels; n=9) Hpa2 expression compared with the primary lesion. In other cases, Hpa2 staining intensity appeared comparable in the primary lesion and metastases (upper panels; n=15). **C**. Kaplan-Meier survival plot. Overall survival analysis was performed for the four groups of the primary (P) and metastasis (M) discordance assay (P^-M^-, P^-M^+, P^+M^-, P^+M^+). Patients exhibiting strong staining of Hpa2 in the primary lesion and the resulting metastasis survive significantly less than the other study groups (p=0.03). **D**. Schematic diagram of the Hpa2 and Hpa2-Nuc gene construct. The signal peptide of Hpa2 (SP; yellow) was removed in the Hpa2-Nuc gene construct, and was replaced by three nuclear localization signal (NLS) repeats introduced at the protein C-terminus (upper panel). Transfection of HEK-293 cells, followed by immunofluorescent staining of Hpa2, showed that Hpa2 localizes in the cell cytoplasm (green, third panels) while Hpa2-Nuc is indeed directed to the cell nucleus (green; lower panel). Nuclei were counter-stained with PI (red). **E**. qPCR. Total RNA was extracted from ZR-75-1 control (Vo), Hpa2, and Hpa2-Nuc cells and was subjected to qPCR analysis applying primer set specific for Hpa2. Hpa2/Hpa2-Nuc expression is presented relative to control (Vo) cells, set arbitrarily to a value of 1 and calculated after normalization to actin.

Suppl. Figure 2. Hpa2 promotes cell motility. Invasion (upper panels) of control (Vo), Hpa2, and Hpa2-Nuc ZR-75-1 cells was examined in Boyden chamber inserts coated with reconstituted ECM (Matrigel). Shown are representative images of the indicated insert at x25 magnification. Quantification of invading cells is shown graphically in the second panel. Wound healing by control (Vo) and Hpa2/Hpa2-Nuc overexpressing ZR-75-1 cells was examined by a quantitative IncuCyte technology (Sartorius). Cells were plated in 96-well plates, grown to confluency, and wound scratches were then performed. The capacity of cells to migrate and fill the wound area was monitored over time. Shown are photomicrographs of the wound (yellow) at the indicated time

points (third panel); quantification of the wound closer is shown in the lower panel. **B**. Increased cell migration by Hpa2 is HS-dependent. Migration of control (Vo) and Hpa2/Hpa2-Nuc MCF10Ca cells was examined by a Boyden chamber assay in the absence (-) or presence (+) of heparin (10 µg/ml; upper panel) or anti-Hpa2 monoclonal antibody 1c7 (30 µg/ml; lower panel) that target the HS-binding domain of Hpa2.

Suppl. Figure 3. Hpa2 promotes, while Hpa2-Nuc attenuates the motility of MDA-MB-231 cells. **A.** qPCR. Total RNA was extracted from control (Vo), Hpa2, and Hpa2-Nuc cells and was subjected to qPCR applying primers specific for Hpa2. Hpa2/Hpa2-Nuc expression is presented relative to control (Vo) cells, set arbitrarily to a value of 1 and calculated after normalization to actin. **B**. Cell motility. Migration (left panels) and invasion (right panels) of control (Vo), Hpa2, and Hpa2-Nuc cells were examined applying fibronectin-coated (migration) and Matrigel-coated (invasion) inserts. Shown are representative images at original magnification of x50. Quantification of migrating and invading cells is shown in the lower panels. **C**. Colony formation. Control (Vo) Hpa2 and Hpa2-Nuc cells were grown in soft agar as described under 'Materials and Methods'. After 5 weeks, dishes were fixed with formalin and cell colonies were stained with Crystal violet. Representative photomicrographs are shown in the upper panels (original magnifications x10). Quantification of the number of colonies per dish is shown graphically in the lower panel.
D. Cell proliferation. Control (Vo), Hpa2, and Hpa2-Nuc cells ($2x10^3$/well) were seeded in a 96-well plate in medium supplemented with 2.5% FCS. At the indicated time points, 20 µl of MTT were added for 3 h and absorbance at 570 nm was measured by a plate reader. Note decreased proliferation of Hpa2-Nuc cells.

Suppl. Figure 4. Hpa2 promotes tumor growth and metastasis. Control (Vo) and Hpa2-over expressing MDA-MB-231 cells ($5x10^6$) were implanted orthotopically in the mammary gland of NOD/SCID mice (n=7). After 4 weeks, tumors were removed under anesthesia, and mice were kept for additional 4 weeks. Mice were then sacrificed and inspected for tumor recurrence (**A**, red arrow) and swollen lymph nodes (**A**; white arrows). 5/7 mice implanted with Hpa2 cells exhibited metastatic lesions also in the opposite mammary gland (**A**, black arrows; **B**),

Suppl. Figure 5. Hpa2 promotes lymph-angiogenesis. 5-micron sections of tumors xenografts produced by control (Vo), Hpa2, and Hpa2-Nuc MDA-MB-231 cells were subjected to immunostaining applying anti-LYVE (a marker of lymphatic endothelial cells). Shown are representative images at original magnification of x25 (upper panels) and x100 (second panels).

Sections were similarly stained for the presence of macrophages, applying anti-F4/80 antibody (third panels) and for Ki67 (indicative of cell proliferation; lower panel). Quantification of Ki67-positive cells is shown graphically in the lower right panels. Shown are representative images at original magnification x100. **B**. qPCR. Total RNA was extracted from the indicated ZR-75-1 cell clone and subjected to qPCR applying primers specific for Hpa2. Hpa2-Nuc expression is presented relative to control (Vo) cell clone (#2), set arbitrarily to a value of 1 and calculated after normalization to actin. Arrows point to Hpa2-Nuc cell clones selected for experimentation.

Suppl. Figure 6. **A-C**. qPCR. Total RNA was extracted from the indicated ZR-75-1 cell clone and subjected to qPCR applying primers specific for IFI44 (**A**), IFI44L (**B**), and CXCL10 (IP10; **C**). **D**. Anoikis. Control (Vo), Hpa2, and Nuc MDA-MB-231 cells ($1x10^6$) were cultured on Poly HEMA-coated dishes in DMEM medium supplemented with 2.5% FCS. Cell viability was quantified after 4 days using Annexin V-FITC apoptosis detection kit. Shown are the original dot plots (upper panels); The percent of apoptotic cells in each group is shown graphically in the lower panel. **E**. qPCR. Total RNA was extracted from ZR-75-1 (ZR), BT-549, SUM 149, MDA-MB-157 (157), MDA-MB-231 (231), and MDA-MB-468 (468) breast carcinoma cells and subjected to qPCR applying primers specific for Hpa2. Hpa2 expression is presented relative to ZR-75-1 cells, set arbitrarily to a value of 1 and calculated after normalization to actin. **F**. Sections of tumors produced by ZR-75-1 Vo (#2, #5, #6) and Hpa2-Nuc (#27, #31) cell clones were subjected to Masson's Trichrome staining. Shown are representative photomicrographs at original magnification of x100.

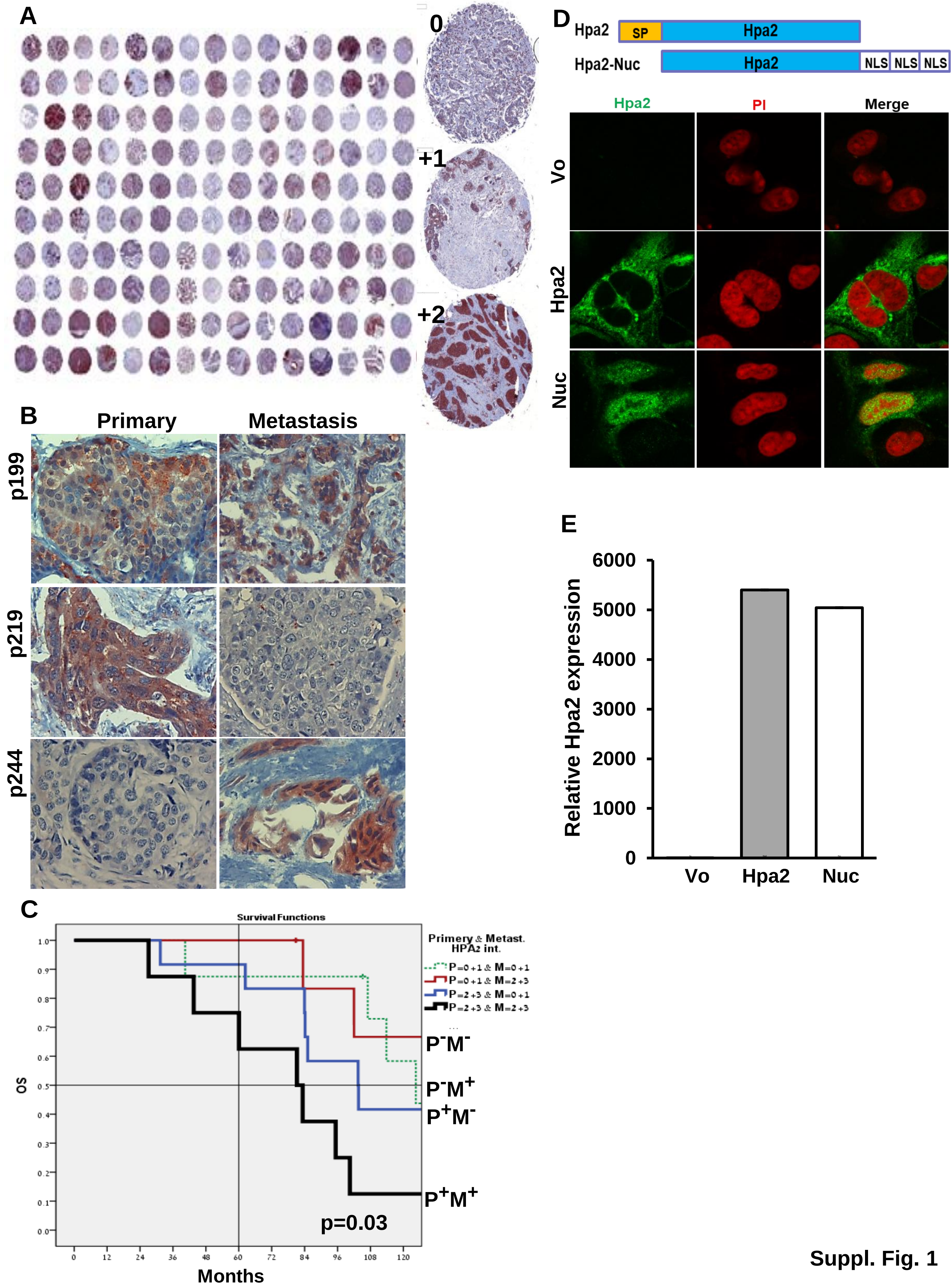
A
0
+1
+2
B
Primary
Metastasis
p199
p219
p244
C
Survival Functions
Primery & Metast. HPA2 int.
P=0+1 & M=0+1
P=0+1 & M=2+3
P=2+3 & M=0+1
P=2+3 & M=2+3
P-M-
P-M+
P+M-
P+M+
p=0.03
OS
Months
D
Hpa2
SP
Hpa2-Nuc
NLS
Hpa2
PI
Merge
Vo
Nuc
E
Relative Hpa2 expression
6000
5000
4000
3000
2000
1000
0
Vo
Hpa2
Nuc

Suppl. Fig. 1

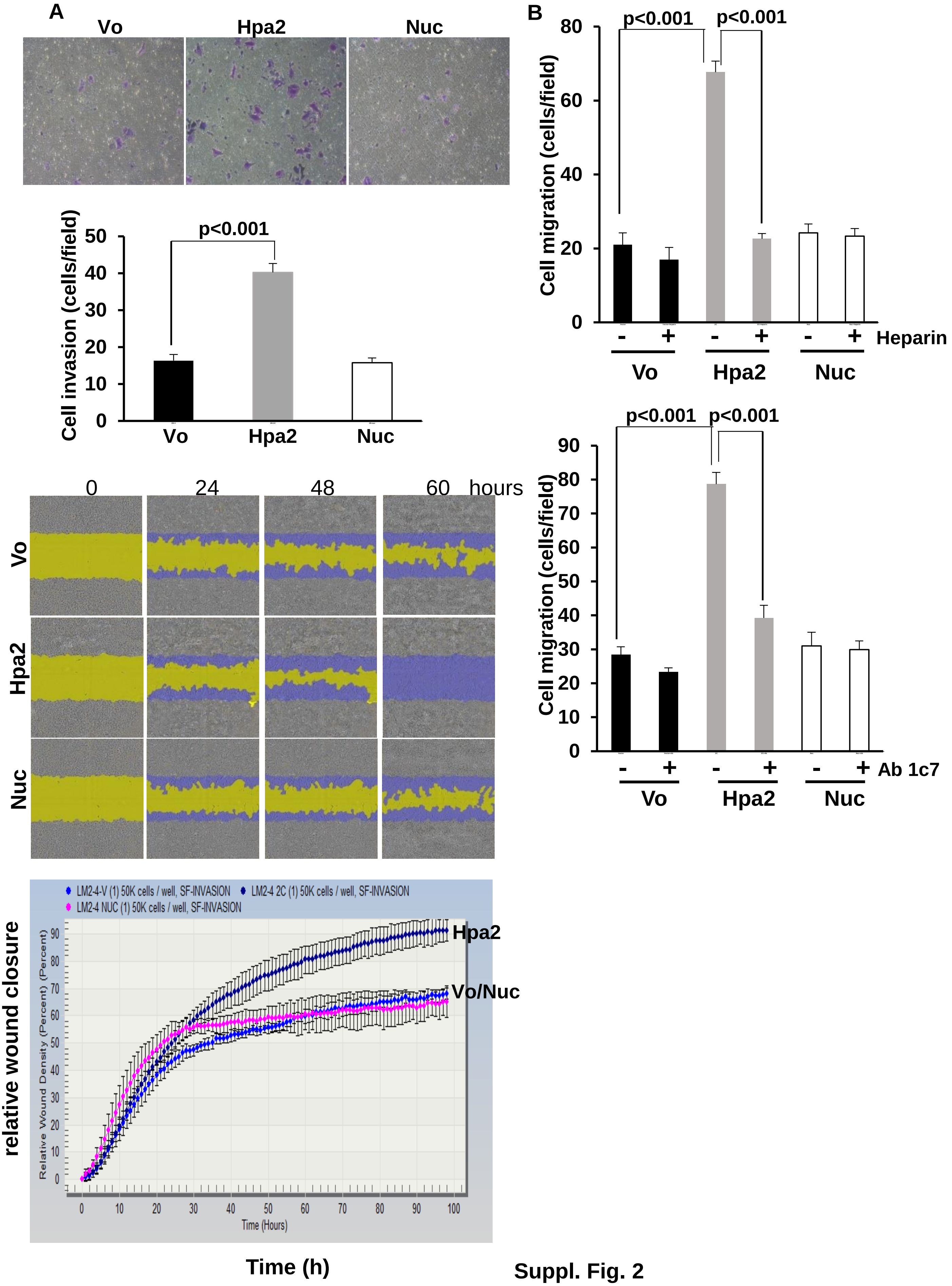

A
Vo
Hpa2
Nuc
p<0.001
Cell invasion (cells/field)
0
10
20
30
40
50
B
p<0.001
p<0.001
Cell migration (cells/field)
0
20
40
60
80
- + - + - + Heparin
Vo
Hpa2
Nuc
p<0.001
p<0.001
90
80
70
60
50
40
30
20
10
0
- + - + - + Ab 1c7
0
24
48
60
hours
LM2-4-V (1) 50K cells / well, SF-INVASION
LM2-4 2C (1) 50K cells / well, SF-INVASION
LM2-4 NUC (1) 50K cells / well, SF-INVASION
Relative Wound Density (Percent) (Percent)
Time (Hours)
Hpa2
Vo/Nuc
relative wound closure
Time (h)

Suppl. Fig. 2

Suppl. Fig. 3

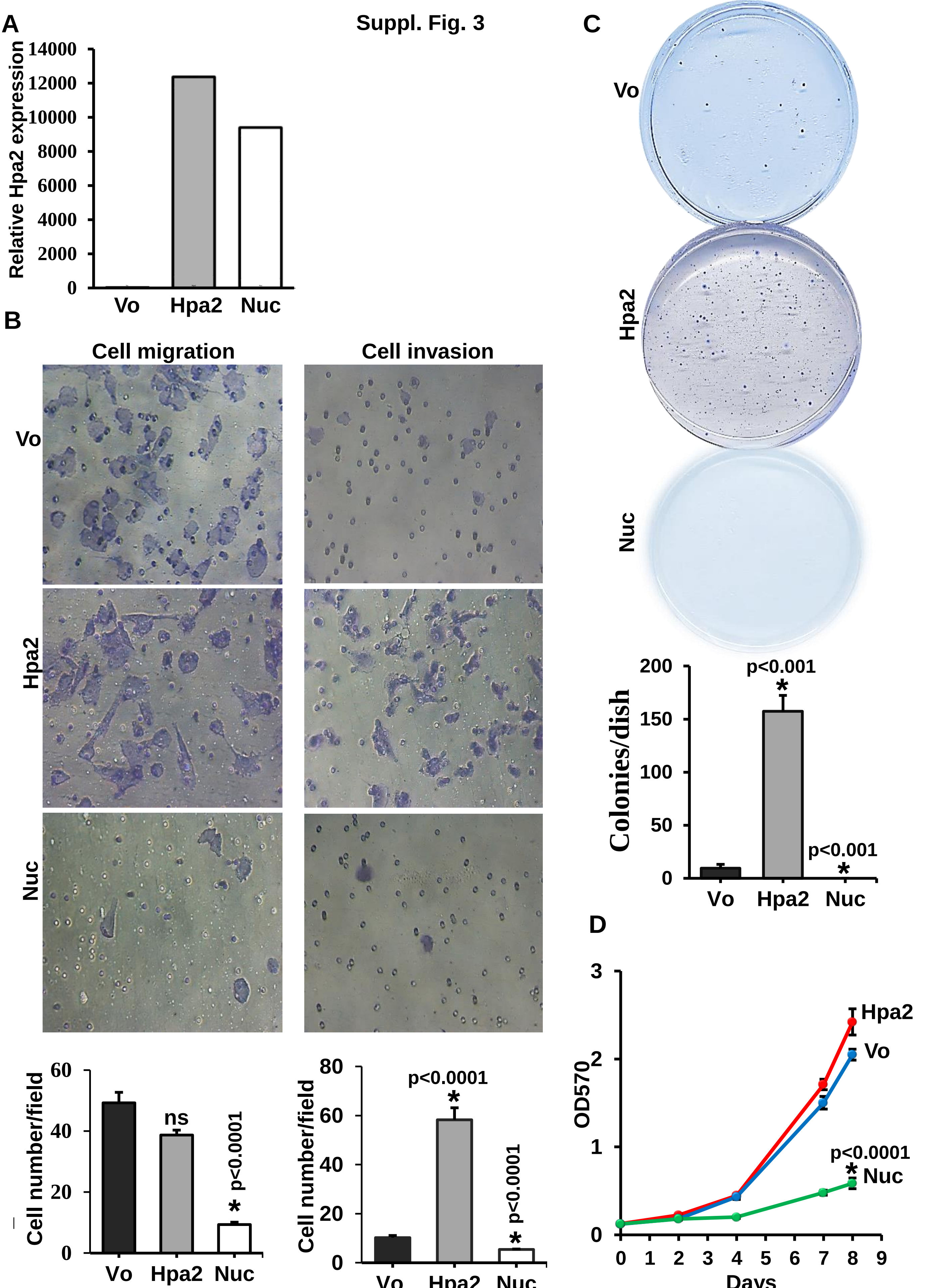
A
Relative Hpa2 expression
14000
12000
10000
8000
6000
4000
2000
0
Vo
Hpa2
Nuc
B
Cell migration
Cell invasion
Vo
Hpa2
Nuc
Cell number/field
60
40
20
0
ns
p<0.0001
*
Vo
Hpa2
Nuc
Cell number/field
80
60
40
20
0
p<0.0001
*
p<0.0001
*
Vo
Hpa2
Nuc
C
Vo
Hpa2
Nuc
Colonies/dish
200
150
100
50
0
p<0.001
*
p<0.001
*
Vo
Hpa2
Nuc
D
OD570
3
2
1
0
Hpa2
Vo
p<0.0001
*
Nuc
0
1
2
3
4
5
6
7
8
9
Days

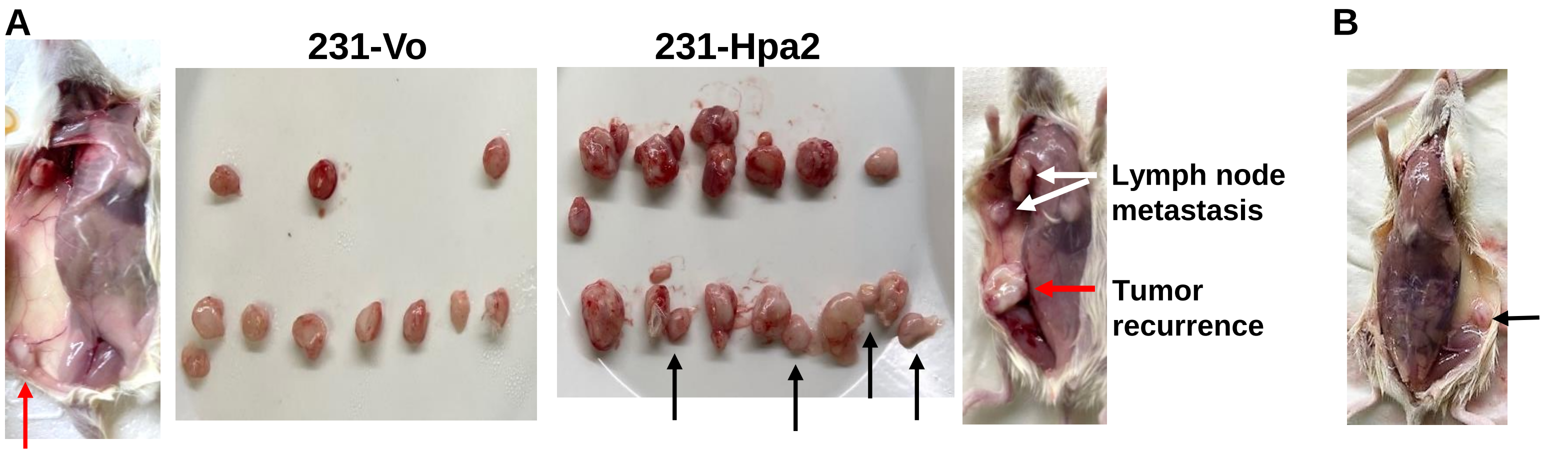

Suppl. Fig. 4

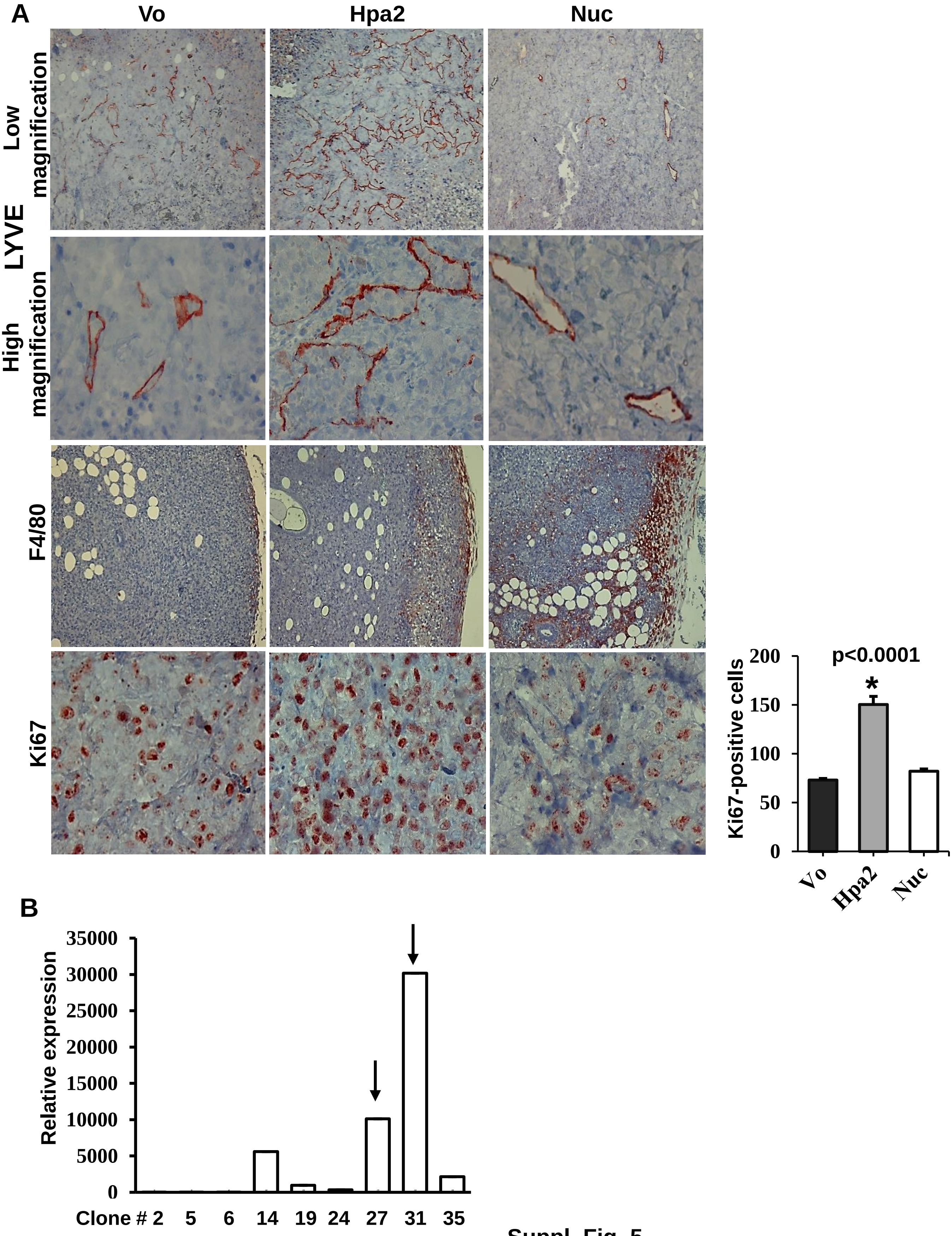
A
Vo
Hpa2
Nuc
Low magnification
LYVE
High magnification
F4/80
Ki67
p<0.0001
*
Ki67-positive cells
200
150
100
50
0
Vo
Hpa2
Nuc
B
Relative expression
35000
30000
25000
20000
15000
10000
5000
0
Clone # 2
5
6
14
19
24
27
31
35

Suppl. Fig. 5

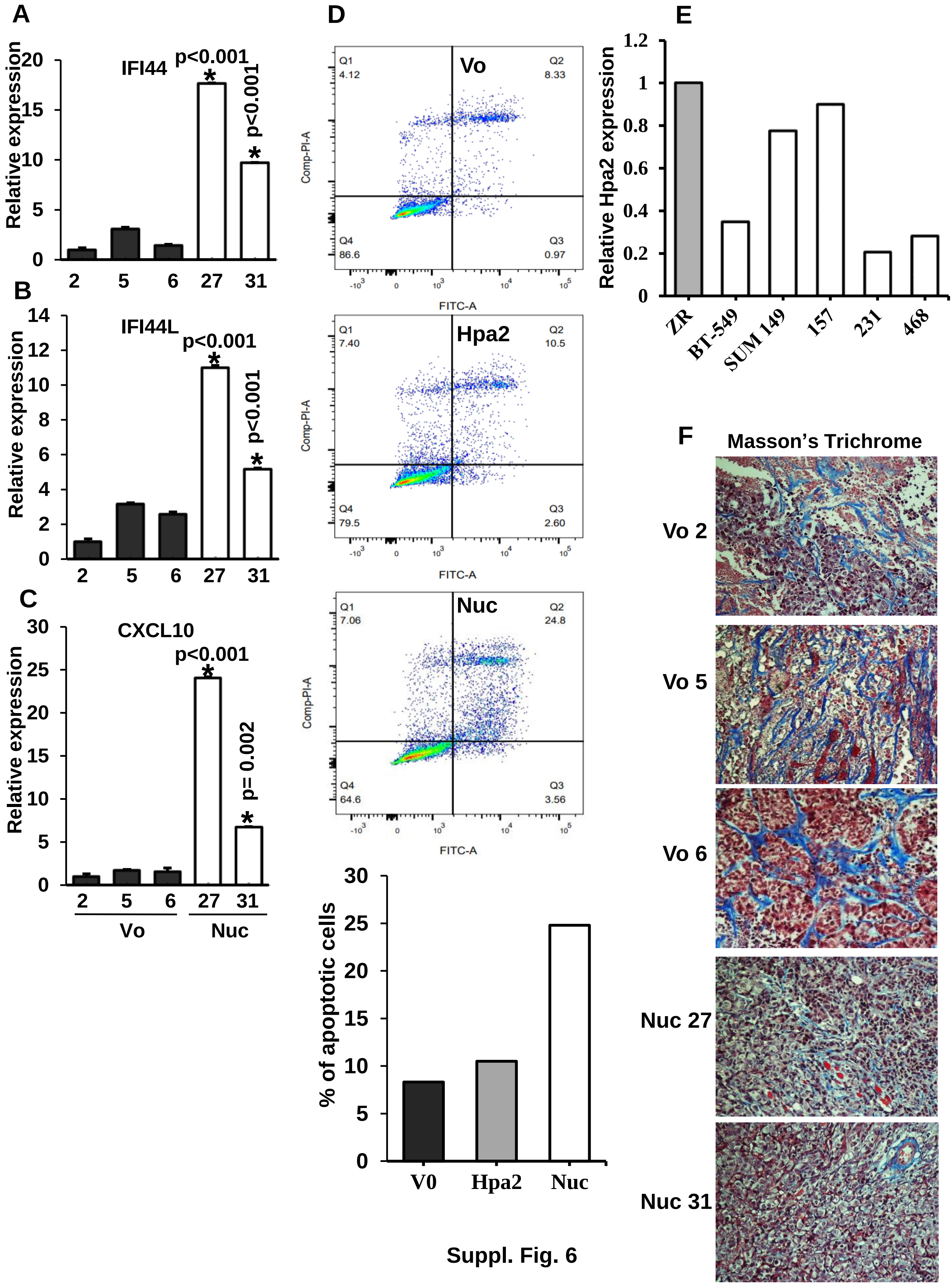
A
IFI44
p<0.001
p<0.001
Relative expression
B
IFI44L
p<0.001
p<0.001
Relative expression
C
CXCL10
p<0.001
p= 0.002
Relative expression
2 5 6 27 31
Vo
Nuc
D
Vo
Hpa2
Nuc
Q1 4.12
Q2 8.33
Q4 86.6
Q3 0.97
Q1 7.40
Q2 10.5
Q4 79.5
Q3 2.60
Q1 7.06
Q2 24.8
Q4 64.6
Q3 3.56
Comp-PI-A
FITC-A
% of apoptotic cells
V0
Hpa2
Nuc
E
Relative Hpa2 expression
ZR
BT-549
SUM 149
157
231
468
F
Masson's Trichrome
Vo 2
Vo 5
Vo 6
Nuc 27
Nuc 31

Suppl. Fig. 6

Suppl. Table 1. Demographic and clinical characteristics of the patients (n=61) enrolled in this study **(first cohort)**

**Age:**	Mean-36.4<br>Median-37.1<br>(19.7-44.95)
**T:**	1-29<br>2-24<br>3-4<br>4-4
**N:**	No-28<br>Yes-31<br>Missing-2
**Stage:**	1-19<br>2-25<br>3-17
**Grade:**	Mod-31<br>Poor-25<br>Missing-5
**Diagnosis at pregnancy:**	No-56<br>Yes-4<br>Missing-1
**Family history of BC:**	No- 42<br>Yes-19
**BRCA**	Negative-9<br>Positive-4<br>Unknown-48
**ER**	Pos- 27<br>Neg- 21<br>Missing- 13
**PR**	Pos- 32<br>Neg- 16<br>Missing- 13
**Hpa2**	Neg- 18<br>Weak (+1)- 14<br>Strong (+2)- 19

Suppl. Table 2. Demographic, clinical,
and molecular characteristics of the patients (n=150)
enrolled in this study (second cohort)

**Age:**	Mean-49<br>33-75
**T:**	1- 2<br>2-96<br>3-36<br>4-16
**N:**	No-120<br>Yes- 30
**Grade:**	1- 10<br>2-106<br>3- 22<br>Missing-12
**Stage:**	I-2<br>IIA- 88<br>IIB- 28<br>IIIA-12<br>IIIB-16<br>IV-4
**AR**	0-42<br>1-32<br>2-50<br>3-25
**ER:**	0-76<br>1-14<br>2-35<br>3-25
**PR:**	0-93<br>1-13<br>2-31<br>3-13
**Ki67:**	0-49<br>1-38<br>2-57<br>3- 6
**Hpa2:**	Weak- 50<br>Strong-96<br>Missing-4

Suppl. Table 3. Primer sets employed in this study

Genes		Sequence (5’-3’)
		**Human**
Hpa2	F:	CAGGGCATTGATGTCGTGATAC
	R:	GCCAGTAGTCTGGTAATGGGTTA
VEGF-A	F:	TCTACCTCCACCATGCCAAGT
	R:	TGTCCACCAGGGTCTCGATT
IFI44	F:	GGTGGGCACTAATACAACTGG
	R:	CACACAGAATAAACGGCAGGTA
IFI44L	F:	ACAGAGCCAAATGATTCCCTATG
	R:	TCGATAAACGACACACCAGTTG
CXCL10	F:	TCCACGTGTTGAGATCATTGC
	R:	TCTTGATGGCCTTCGATTCTG
		**Mouse**
NK1.1	F:	GCTGTGCTGGGCTCATCCT
	R:	TTGATGGTTTTTGTACTAAGACTCGCA
Granzyme	F:	TGTCTCTGGCCTCCAGGACAA
	R:	CTCAGGCTGCTGATCCTTGATCGA
